# Supplementary material for: Development and Implementation of Dried Blood Spot-Based COVID-19 Serological Assays for Epidemiologic Studies
Source: Microbiol Spectr. 2022 May 25;10(3):e02471-21. doi: 10.1128/spectrum.02471-21 (PMC9241704; doi:10.1128/spectrum.02471-21)
Supplement: SUPPLEMENTAL FILE 1 — Fig. S1 to S4. Download spectrum.02471-21-s001.pdf, PDF file, 0.2 MB [file spectrum.02471-21-s001.pdf]

Figure S1

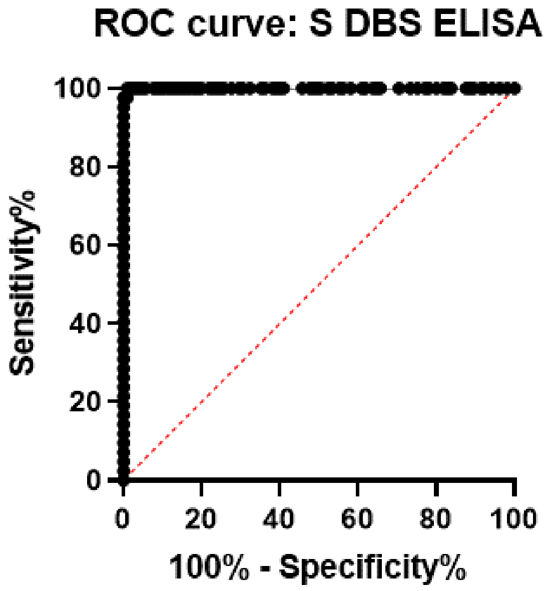

Supplemental Figure 1. Receiver-Operator Curve (ROC) of the validation of the anti-S IgG DBS ELISA.

Figure S2

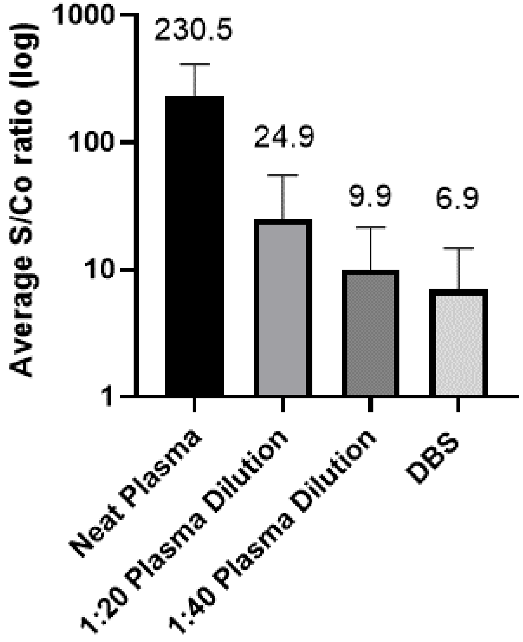

Supplemental Figure 2. Comparison of diluted plasma to DBS on the Ortho COV2T assay. Paired plasma-DBS samples (n=37) were tested undiluted or at the indicated dilutions of plasma. Numbers indicate the average S/Co ratio; error bars represent standard deviation.

Figure S3

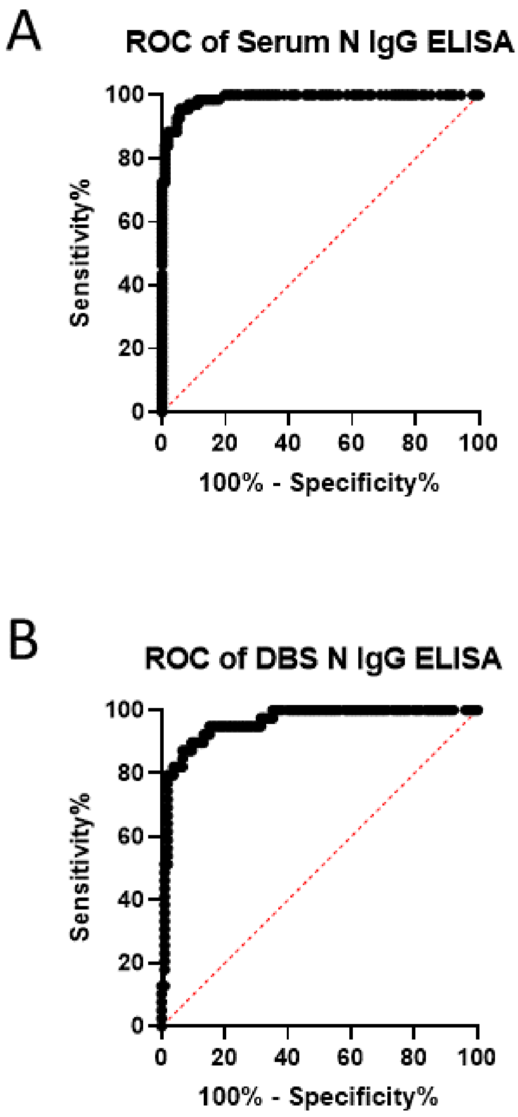

Supplemental Figure 3. Receiver-Operator Curves (ROC) of the validation of (A) the anti-N IgG ELISA in serum or of (B) the anti-N DBS ELISA.

Figure S4

A

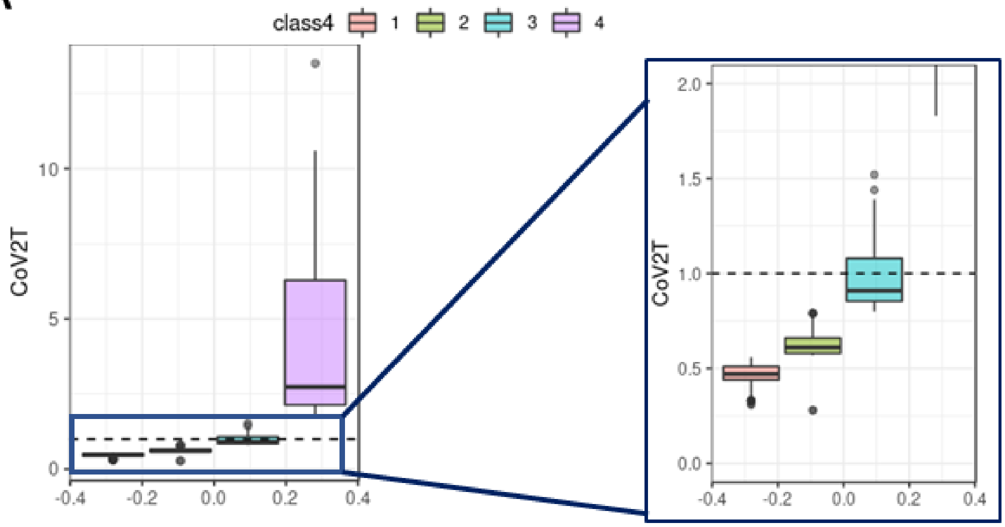

B

	N	% of Total	Mean S/Co	SD
Class 1	3753	85.88%	0.472	0.0471
Class 2	559	12.79%	0.626	0.0586
Class 3	47	1.08%	0.997	0.195
Class 4	11	0.25%	4.87	4.08
Total	4370	100%		

Supplemental Figure 4. (A) Ortho COV2T results from Round 1 of the EBCOVID study grouped into 4 categories. Right panel is an inset of the boxed area in the left panel. Dotted line represents the positivity cut-off value. (B) Descriptive table of data in (A). Highlighted row refers to results that fall within the indeterminate zone.
